# Thyroid Dysfunction and Cytological Patterns among Patients Requested for Thyroid Function Test in an Endemic Goiter Area of Gondar, North West Ethiopia

**DOI:** 10.1155/2019/9106767

**Published:** 2019-08-14

**Authors:** Daniel Asmelash, Kumlgn Tesfa, Belete Biadgo

**Affiliations:** ^1^Department of Clinical Chemistry, College of Medicine and Health Science, University of Gondar, Gondar, Ethiopia; ^2^Department of Medical Laboratory Science, University of Gondar, Gondar, Ethiopia

## Abstract

**Background:**

Thyroid dysfunction is the most common endocrine disorder in clinical practice, and about half of the population with thyroid dysfunction remains undiagnosed. There is a fairly wide spectrum of thyroid dysfunction, which can be identified by patterns of thyroid function test results. The prevalence of thyroid dysfunction among the population varies in different studies.

**Methods:**

A cross-sectional study was conducted from February 8th to April 8th, 2017, among patients who requested for the thyroid function test in an endemic goiter area at the Gondar Hospital, University of Gondar. A pretested structured questionnaire was used to collect the data. Three milliliters of blood samples was collected in a plain test tube and centrifuged for serum separation. The thyroid function test was done by using the MINI-VIDAS automation following the manufacturer manual (Setema PLC, Italy). Data were entered and analyzed using SPSS version 20. Descriptive statistics were used for data presentation, and *P* value < 0.05 was considered significant.

**Result:**

Of the total 384 study participants, 346 (90.1%) were females and the study participants' mean age was 38 ± 13.9 years. The overall thyroid dysfunction prevalence was 26.3% (101): 1.6% was identified as subclinical hypothyroidism, 0.5% hypothyroidism, 9.6% subclinical hyperthyroidism, and 14.6% hyperthyroidism, and 23.4% had goiter. Furthermore, for cytological pattern analysis, 144 study participants who fulfilled indications for fine-needle aspiration cytology (FNAC) in thyroid nodules were included. Of the total, 3 (2.1%) had thyroid carcinoma, 46 (32%) had cystic degenerated follicular cells, and 82 (57%) had nodular thyroid goiter. In addition, a clinical presentation of a total of 144 study participants, showed lymphadenites in 7 participants (4.8%), hypertension in 9 (6.2%), and cardiac failure in 12 (8.3%).

**Conclusion:**

The prevalence of thyroid dysfunction was high. The majority of thyroid dysfunction cases were newly diagnosed and more common in females. In addition, the most common disorders were subclinical hyperthyroidism and hyperthyroidism. Follicular cell with cyst degeneration and thyroid nodular goiter were the predominant FNAC findings. For early diagnosis and appropriate intervention in goiter endemic areas, the thyroid function test should be closely monitored.

## 1. Introduction

Thyroid dysfunction is described as the altered serum thyroid-stimulating hormone (TSH) level with normal or altered thyroid hormones. Thyroid dysfunction is a major public health problem, and the prevalence of thyroid dysfunction depends on environmental factors, ethnic, and iodine intake status [1, 2].

Hypothyroidism and hyperthyroidism are two widespread thyroid problems, of which hypothyroidism is much more common. In its clinical form, hypothyroidism is a relatively common condition, with an approximate prevalence of 2% in adult women and 0.2% in adult men [3]. The clinical features of hypothyroidism are dependent on the patient's age, the presence of other diseases, and the rate at which hypothyroidism develops [4].

Significant risk factors for thyroid dysfunction, specifically hypothyroidism and subclinical hypothyroidism, are smoking, family history of thyroid disease, female gender, alcohol, pregnancy, age, body mass index (BMI), family history of diabetes mellitus, and iodine intake [5]. Higher risks for the developing of hypothyroidism are autoimmune disease, women with postpartum period, personal history of neck or head irradiation, primary pulmonary hypertension, genetic syndromes, and people over 65 years old [6].

The effect of subclinical hyperthyroidism and hypothyroidism in the general population causes cardiovascular-related morbidity and mortality [7]. Thyroid dysfunctions such as hypothyroidism and thyrotoxicosis can affect the health of both the mother and the child before and after delivery, which can lead to fetal disease in humans, including a high incidence of mental retardation. It can occur in about 1% of the population and up to 0.4% of pregnancies [8]. The psychiatric disturbances that accompany hyperthyroidism and hypothyroidism mimic mental illness [9].

Thyroid dysfunction is a common endocrine disorder affecting around 300 million people worldwide and it is presumed that more than half are unaware of their condition. The major thyroid disorders are hyperthyroidism and hypothyroidism, with 1.6 billion people at risk in more than 110 countries around the world [10]. The prevalence of hypothyroidism in the US population was 4.6%, but there is a clinically evident hypothyroidism in 0.3%. Women are 5 to 8 times more likely to have thyroid problems than men. In addition, one in eight women during her lifetime will develop a thyroid disorder [6, 11].

The most prevalent thyroid diseases in Africa include hypothyroidism, thyrotoxicosis, thyroiditis, and iodine deficiency disorders. Iodine deficiency is a common cause of thyroid disorder and a major public health problem across Africa. A study conducted among immigrants in Ethiopia showed that the goiter prevalence was 46.1%, the prevalence of hyperthyroidism 1 was .7%, and the prevalence of hypothyroidism was 1.1% [12–14].

The causes of thyroid nodules are iodine deficiency, thyroid adenomas (autonomous or hyperfunctional thyroid nodules) and thyroid cyst. Thyroid cancer and nodules are common and can occur in up to 60% of the population. Fine-needle aspiration cytology (FNAC) in thyroid nodules has higher sensitivity and is a rapid, cost-effective, and very useful method for classifying thyroid nodules as either benign nodules, reducing unnecessary surgery, or malignant nodules requiring surgery. There have been several guidelines or indications on when to perform FNAC in thyroid nodules. [15, 16].

Thyroid dysfunction affects a considerable portion of the population in Africa including Ethiopia. However, there is a limited information on thyroid dysfunction and cytological pattern in our study area. Therefore, this study tried to find evidence-based data on the prevalence of thyroid dysfunction and cytological pattern in Gondar, Ethiopia.

## 2. Methods and Materials

### 2.1. Study Design, Study Area, and Period

A cross-sectional study was conducted on patients requested for the thyroid function test (TFT) at the University of Gondar Hospital. The study was carried out among patients clinically suspected of thyroid disorder and requested from February 8 to April 8, 2017, at the University of Gondar Hospital.

### 2.2. Sample Size and Dependent and Independent Variables

A total of 384 study participants were included by using simple random sampling technique. Dependent and independent variables were thyroid dysfunction, age, gender, religion, marital status, residence, thyroid medication, family history, pregnancy, and iodinated salt intake.

### 2.3. Inclusion and Exclusion Criteria

Patients requested for the thyroid function test (TFT) and willing to participate in the study were included using simple random sampling technique. In addition, a total of 144 study participants who fulfilled guidelines or indications on when to perform FNAC [16] in thyroid nodules were included for cytological pattern analysis. However, critically ill patients, who were unable to communicate, and pregnant women were excluded from the study.

### 2.4. Data Collection and Laboratory Methods

Sociodemographic and clinical data were collected by the investigator at the University of Gondar Hospital by using a semistructured questionnaire. Three milliliters of the blood samples was collected in a plain test tube, allowed to clot, and centrifuged at 3,000 rpm for 15 minutes for serum separation by medical laboratory science professional for thyroid function test determination. Patient serum samples were tested within 1 hour of sample collection, and thyroid function tests T3 (nmol/l), T4 (nmol/l), and TSH (*μ*I*μ*/ml) were estimated using an automated immunoassay analyzer (MINI-VIDAS (Setema PLC, France)). In addition, a total of 144 study participants who fulfilled guidelines or indications for FNAC in thyroid nodules were investigated at the University of Gondar Hospital's pathology department.

### 2.5. Data Management and Quality Control

Before actual data collection, the questionnaire was tested for its accuracy and consistency. Appropriate information was provided to data collectors regarding the study objective and relevance, confidentiality issues, the right of the study participants, consent, interview techniques, and laboratory test procedures and quality control. The laboratory tests were performed by two senior medical laboratory scientists. In addition, FNAC was done by the senior pathologist at the University of Gondar Hospital. The data collected were carefully checked for completion, accuracy, and clarity. A laboratory test was analyzed after running the quality control sample, and the method was ensured to be safe. The laboratory's quality control was evaluated using the University of Gondar Hospital laboratory manuals and SOPs (standard operating procedures). In addition, preanalytical, analytical, and postanalytical quality precautionary measures depending on the stated SOP were considered to maintain the quality of the result.

### 2.6. Data Analysis and Interpretation

The data were collected by interviewing patients and checked, sorted, categorized, and coded manually. Finally, the data were entered and analyzed using SPSS version 20. The data were analyzed to determine the frequencies of different thyroid dysfunction categories. In addition, the data were classified into subgroups by age and sex to determine the association between age and sex with thyroid dysfunction. The relative frequencies and ratios of each category of thyroid dysfunction and clinical and cytological patterns were determined. A chi-squared test was performed, and a *p*-value of <0.05 was considered to be statistically significant. Frequency bar charts and tables were prepared using the Microsoft Excel software program.

### 2.7. Ethical Considerations

Ethical clearance was obtained from the Research and Ethical Review Committee of the School of Biomedical and Laboratory Sciences, College of Medicine and Health Sciences, University of Gondar. Permission letter was also taken from the clinical director of the hospital and head of the clinical chemistry laboratory. The data were collected after full written consent was obtained from each participant. All the study participants were informed about the purpose of the study, and finally, written consent was obtained before the data collection.

## 3. Results

### 3.1. Sociodemographic Characteristics of the Study Participants

This study included a total of 384 study participants requested for the thyroid function test. Of the total study participants, 346 (90.1%) were females, 319 (83.1%) were illiterate, and 25.8% were in the age group between 25 and 35 years. In addition, the mean age of the study participants was (mean ± SD) 38.05 ± 13.93 (Table 1).

The overall prevalence of thyroid dysfunction among the study participants was 26.3% (101) (95% CI: 0.22–0.31). Of the total thyroid dysfunction, the majority was in the 36- to 45-year age group (29 (7.5%)) and the majority was newly diagnosed (83.2%). In addition, their clinical presentation showed that 43 (11.2%) had additional disease comorbidities, 90 (23.4%) had goiter, and 35 (9.1%) had a family history of thyroid disorder (Table 2).

### 3.2. Prevalence of Thyroid Dysfunction among the Study Participants

**Figure 1:**
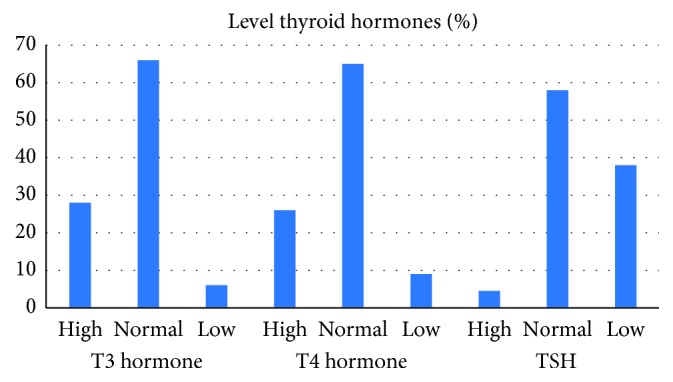
Levels of thyroid hormone among participants.

Of the total, 108 participants (28.1%) had high T3, 101 (26.3%) participants had high T4, and 17 (4.4%) participants had high TSH. The mean concentration of T3, T4, and TSH was 2.35, 110.6, and 1.69, respectively (Figure 1).

**Figure 2:**
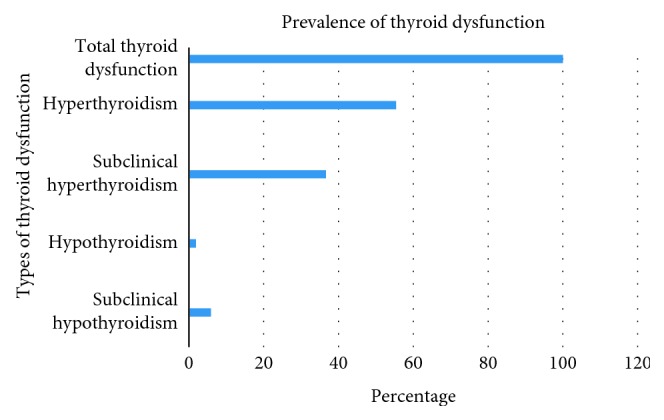
Prevalence of thyroid dysfunction patterns among the study participants.

The overall prevalence of thyroid dysfunction among the study participants was 101 (26.3%) (95% CI: 0.22–0.31). Of all thyroid dysfunction, subclinical hyperthyroidism and hyperthyroidism were 36.6% and 55.4%, respectively (Figure 2).

### 3.3. Thyroid Dysfunction by Age and Sex of the Study Participants

Thyroid dysfunction was present in 26.3% of the total participants. Females had a higher prevalence of thyroid dysfunction than males in all types of thyroid dysfunction. In addition, the majority of subclinical hypothyroidism (14, 37.8%) and hyperthyroidism (14, 25%) were in the 36- to 45-year age group (Table 3).

### 3.4. Cytological Pattern and Clinical Data of the Study Participants

From total study participants, only 144 (37.5%) were fulfilled with the indication for FNAC investigation. Of the total, the major subtypes of benign lesions were the colloid goiter and thyroid cystic degeneration. Furthermore, colloid goiter and thyroid cyst degeneration predominated in females and in the age group of 36–45 years. Moreover, lymphadenites, hypertension, and cardiac failure were predominant among females and in the 36- to 45-year-old study participants. Of the total clinical presentation, cardiac failure and hypertension were accounted for the majority (Table 4).

## 4. Discussion

In this study, the overall prevalence of thyroid dysfunction was 26.3% among 384 participants. Our study results showed higher values than the results of the studies from southern Nepal (25%) [17], western Nepal (17.42%) [18], and Uganda (3.6%) [19]. Reasons for the variation in the prevalence of thyroid dysfunction may be due to the difference in study population sociodemographic characteristics.

The prevalence of hypothyroidism in this study was 0.5%. This finding was lower compared to that from the studies performed in southern Nepal (8.9%) [17], Philippines (0.41%) [20], (10.95%) [21], southern India (11%) [22], and western Nepal (2.6%) [18].

The prevalence of subclinical hypothyroidism in this study was 1.6%. This finding was lower compared to that in the studies from southern Nepal (2.5%) [17], Philippines (2.18%) [20], Brazil (6.5%) [23], western Nepal (10.5%) [18], Saudi Arabia (19%) [24]. The difference in the result may be due to the difference in sample size, study population, and methods of thyroid hormone analysis.

The prevalence of hyperthyroidism in this study was 14.6%. This result of our study was higher compared to that of studies from Nepal (8.9%) [17], Cochin, India, (1.3%) and South India (1.3%) [22], Norway (3.1%) [25], western Nepal (1.5%) [18], and Saudi Arabia (0.7%) [24]. The overall prevalence of subclinical hyperthyroidism was 9.6%. The result of this study was higher than that of Cochin institutional-based study, India (1.6%) [22], Brazil (5.4%) [23], western Nepal (3.05%) [18], and Saudi Arabia (2.6%) [24]. The difference in the result may be due to difference in levels of iodine deficiency and genetic factors.

In this study, the prevalence of goiter was 71%. The result was higher compared to that in studies performed in India (39%) [22], Metekel Zone, Ethiopia (8%) [26], and University of Gondar Hospital (50%) [27]. This high prevalence of goiter in our study area may be due to low practice of iodine salt. However, the variation in the findings may be because our study setting was an endemic goiter area.

The overall prevalence of thyroid dysfunction in female participants was 95 (24.7%). This is in agreement with a study report in the US that showed women are five to eight times more likely than men to have thyroid problems. In addition, one in eight women during her lifetime will develop a thyroid disorder [11]. Females were more affected than males in this study. The prevalence of thyroid dysfunction in females and males was 24.7% and 1.6%, respectively. This result was higher than that of the studies from Brazil with 2.8% females [23] and 2.4% males and Norway with 2.5% females and 0.6% males [25]. The variation in the result between males and females may be due to hormonal differences.

In this study, the prevalence of thyroid dysfunction was higher in the 36- to 45-year age group. This is different from the US study; the prevalence was the highest in the age group over the age of 65 years [6]. This may be due to differences in the sociodemographic factors.

Furthermore, the prevalence of newly diagnosed thyroid dysfunction in our study was 22.1% (84) which was higher than the pooled prevalence of undiagnosed thyroid dysfunction in Europe (6.71%) (pooled estimate) (95% CI, 6.49%–6.93%) [28]. This may be because our study setting was an endemic goiter area, but the pooled estimate of Europe was calculated in the general population. Moreover, this may be due to a limited health service and a difference in educational status among the study population.

Of the total participants, 27.3% had a family history of thyroid disorder. This result is in agreement with a study from the University of Gondar Hospital, which showed that family history of thyroid disorder was reported in 26.3% of the study participants [27].

The most common lesion in this study was the colloid goiter and thyroid cystic degeneration as seen in 82 (57%) and 46 (32%), respectively. Different studies have convinced that a benign thyroid lesion is the predominant lesion in the developing world over other types of lesion, including sub-Saharan African countries [29]. The result of this study is consistent with studies conducted in India where 33% of the most common thyroid lesions were simple colloid goiter, 16.28% nodular colloid goiter, and 27.6% goiter with cystic changes [30]. However, this minor variation in result may be caused by the difference in the prevalence of diseases and the clinical criteria for FNAC performance. In addition, in this study, colloid goiter and thyroid cystic degeneration were predominant in females and in the 36- to 45-year age group. This study result is inconsistent with that of the study performed in India, Udaipur [31].

## 5. Conclusion

The study showed that there was a high prevalence of thyroid dysfunction, and the most common disorders were hyperthyroidism and subclinical hyperthyroidism. The majority of thyroid dysfunctions were newly diagnosed and was more common in females. Colloid goiter and thyroid cystic degeneration were the major subtypes of benign lesions and predominated in women and in the 36- to 45-year age group. Moreover, lymphadenites, hypertension, and cardiac failure were predominant among females and in the 36- to 45-year age group of the study participants. Therefore, targeting the control and prevention strategy using iodine supplementation and through health promotion measures towards iodinated salt intake may contribute to the reduction of the prevalence and complications of thyroid disorder.

## Figures and Tables

**Table 1:** Sociodemographic characteristics of the study participants.

Variables	Category	Frequency (*n*)	Percentage (%)
Age (years)	<25	73	19.0
	25–35	99	25.8
	36–45	91	23.7
	46–60	102	26.6
	>60	19	4.9

Sex	Male	38	9.9
	Female	346	90.1

Residence	Rural	94	24.5
	Urban	290	75.5

Educational status	Illiterates	319	83.1
	Elementary school	15	3.9
	Secondary school	10	2.6
	Diploma	32	8.3
	University degree and above	8	2.1

Religion	Orthodox	274	71.2
	Muslim	79	20.5
	Protestant	31	8.3

Marital status	Single	118	30.6
	Married	206	53.6
	Widowed/divorced	62	16.1

**Table 2:** Prevalence of thyroid dysfunction according to sociodemographic and clinical characteristics of the study participants.

Variables	Category	Total *N* (%)	Euthyroid *N* (%)	Thyroid dysfunction *N* (%)	Pearson chi-squares (*χ*^2^)
Age (years)	<25	73 (19)	49 (12.8)	24 (6.2)	0.277
	25–35	99 (25.8)	83 (21.6)	16 (4.2)	
	36–45	91 (23.7)	62 (16.1)	29 (7.5)	
	46–55	81 (21.1)	61 (15.9)	20 (5.2)	
	>55	40 (10.4)	28 (7.3)	12 (3.2)	

Sex	Male	38 (9.9)	32 (8.3)	6 (1.6)	0.121
	Female	346 (90.1)	251 (65.4)	95 (24.7)	

Previously diagnosed (thyroid dysfunction)	Yes	17 (4.4)	1 (0.26)	16 (4.1)	0.032^*∗*^
	No	367 (95.6)	223 (58.1)	84 (22.1)	

Disease comorbidities	Yes	130 (33.9)	87 (22.7)	43 (11.2)	0.031^*∗*^
	No	254 (66.1)	196 (51)	58 (15.1)	

Presence of goiter	Yes	273 (71)	183 (47.6)	90 (23.4)	0.932
	No	111 (29)	100 (26)	11 (2.8)	

Treated for thyroid dysfunction	Yes	31 (8.1)	18 (4.7)	12 (3.1)	0.176
	No	353 (91.9)	264 (68.8)	89 (23.2)	

Family history of thyroid dysfunction	Yes	105 (27.3)	70 (18.2)	35 (9.1)	0.035^*∗*^
	No	279 (72.7)	213 (55.5)	65 (16.9)	

Pregnancy history in the last two years	Yes	61 (15.9)	39 (10.2)	22 (5.7)	0.12
	No	323 (84.1)	244 (63.7)	79 (20.6)	

Iodinated salt practice	Yes	41 (10.7)	27 (7)	13 (3.4)	0.153
	No	343 (89.3)	256 (66.7)	88 (23)	

Duration of illness (year)	<5	337 (87.8)	247 (64.3)	90 (23.4)	0.63
	≥5	47 (12.3)	36 (9.4)	11 (2.9)	

^*∗*^Significant association.

**Table 3:** Spectrum of thyroid dysfunction types according to age and sex of the study participants (*n* = 384).

Variables	Category	Subclinical hypothyroidism		Hypothyroidism		Subclinical hyperthyroidism		Hyperthyroidism	
		*N* (%)	*χ * ^2^	*N* (%)	*χ * ^2^	*N* (%)	*χ * ^2^	*N* (%)	*χ * ^2^
Age (years)	<25	2 (33.3)	0.122	1 (50)	0.294	10 (27.0)	0.223	11 (19.6)	0.044^*∗*^
	25–35	1 (16.7)		0 (0)		5 (13.5)		10 (17.9)	
	36–45	1 (16.7)		0 (0)		14 (37.8)		14 (25)	
	46–55	2 (33.3)		1 (50)		6 (16.2)		11 (19.6)	
	≥56	0 (0)		0 (0)		2 (5.4)		10 (17.9)	

Sex	Male	0 (0)	0.413	0 (0)	0.638	2 (5.4)	0.123	4 (7.1)	0.793
	Female	6 (100)		2 (100)		35 (94.6)		52 (92.8)	

*χ* ^2^ = Pearson chi-square; ^*∗*^significant association.

**Table 4:** Clinical and cytological pattern in relation to age and sex among patients requested for the thyroid function test in an endemic goiter area in 2017 (*n* = 144).

Variables	Sex		Age group (year)				
	Category *N* (%)		Category *N* (%)				
	Male	Female	<25	25–35	36–45	46–55	≥56
*Cytological findings*							
Colloid goiter	14 (9.7)	68 (47.2)	11 (7.6)	27 (18.7)	22 (15.3)	16 (11.1)	16 (11.1)
Cystic thyroid nodules	8 (5.5)	38 (26.4)	4 (2.7)	5 (3.4)	15 (10.4)	12 (8.3)	10 (6.9)
Malignant (follicular)	0 (0)	3 (2.1)	1 (0.7)	0 (0)	0 (0)	2 (1.3)	1 (0.7)
Lymphadenites	0 (0)	7 (4.8)	0 (0)	2 (1.3)	3 (2.1)	1 (0.7)	1 (0.7)

*Clinical presentations*							
Hypertension	1 (0.7)	8 (5.5)	2 (1.3)	0 (0)	6 (4.1)	1 (0.7)	0 (0)
Cardiac failure	4 (2.7)	8 (5.5)	1 (0.7)	1 (0.7)	3 (2.1)	3 (2.1)	4 (2.7)
Diabetes mellitus	1 (0.7)	4 (2.7)	0 (0)	2 (1.3)	1 (0.7)	1 (0.7)	1 (0.7)

## Data Availability

The data generated or analyzed during this study were included in this published article.

## Abbreviations

BMI: Body mass index
DM: Diabetics mellitus
FNAC: Fine-needle aspiration cytology
SCH: Subclinical hypothyroidism
T4: Thyroxine
T3: Triiodothyronine
TFT: Thyroid function test
TSH: Thyroid-stimulating hormone.
